# The copycat phenomenon after two Finnish school shootings: an adolescent psychiatric perspective

**DOI:** 10.1186/1471-244X-12-91

**Published:** 2012-07-28

**Authors:** Nina Lindberg, Eila Sailas, Riittakerttu Kaltiala-Heino

**Affiliations:** 1 Department of Adolescent Psychiatry, Helsinki University Central Hospital, PO Box 590, 00029 HUS Helsinki, Finland; 2 Kellokoski Hospital, 04500 Kellokoski, Finland; 3 Department of Adolescent Psychiatry, University of Tampere, Vanha Vaasa Hospital and Tampere University Hospital, 33380, Pitkäniemi, Finland

## Abstract

**Background:**

Two school shootings with altogether 18 victims took place in Finland in November 2007 and September 2008. Homicides and suicides are both associated with the copycat phenomenon. The aim of the present study was to characterize adolescent copycats who had threatened to carry out a school massacre.

**Methods:**

The nation-wide study evaluated 77 13- to 18-year-old adolescents who were sent for adolescent psychiatric evaluations between 8.11.2007 and 30.6.2009, one of the reasons for evaluation being a threat of massacre at school. The medical files of the copycats were retrospectively analysed using a special data collection form. Data on demographics, family- and school-related issues, previous psychiatric treatment and previous delinquency, current symptoms, family adversities and psychiatric diagnoses were collected. The severity of the threat expressed and the risk posed by the adolescent in question were evaluated. The Psychopathy Checklist Youth Version was used to assess psychopathic traits.

**Results:**

All of the copycats were native Finns with a mean age of 15.0 years. Almost two thirds of them had a history of previous mental health treatment before the index threat. Almost two thirds of the copycats suffered from anxiety and depressive symptoms, and almost half of the sample expressed either suicidal ideation or suicidal plans. Behavioural problems including impulse control problems, aggressive outbursts, the destruction of property as well as non-physical and physical violence against other persons were common. The diagnosis groups highlighted were behavioural and emotional disorders, mood disorders as well as schizophrenia-related disorders. The prevalence of pervasive developmental disorders was high. Only one of the copycats was assessed as expressing high traits of psychopathy.

**Conclusion:**

The copycats with school massacre threats were characterized with a high prevalence of mental and behavioural disorders. Like actual school shooters, they showed psychotic symptoms and traumatic experiences, but unlike the shooters, the copycats were not psychopathic.

## Background

Two school shootings that together resulted in 18 victims took place in Finland in November 2007 and September 2008. Both offenders were young males who used a gun and attempted to burn down the school buildings. They both idealized earlier shooters and used the Internet to document their positive thoughts and ideas on violence as well as videos and statements about their future intentions. Both shootings were characterized by careful planning and ceremonial violence with special clothing and weapons [1], and both differed completely from typical Finnish homicides, which are impulsive manslaughters committed by marginalized boys and men and characterized by alcohol intoxication [2]. This ceremonial type of crime with documentation and sharing it with other persons also differed from traditional premeditated murders and received a great deal of media attention in Finland.

Homicides and suicides are both associated with the copycat phenomenon [3,4]. After the Columbine High School massacre, threats to carry out school shootings increased rapidly, with 354 threats recorded over the next 50 days, far exceeding the one or two threats per year estimated by school administrators before the year 1999 [5]. This phenomenon also happened in Finland. Before the incident in November 2007, threats to carry out a massacre warranting a police investigation were rare, with about 5-10 threats per year, as continues to be the case in the other Nordic countries. After the school shooting in November 2007, and before the second incident, 87 threats were recorded by the police, and within a few weeks from the second incident, more than a hundred new threats were communicated. Still in 2011, the annual number of threats seems to be approaching 60 cases (police statistics, personal communication from Savolainen, M.).

School shootings are not, however, a globally new phenomena, as they date back to at least 1974 [6]. The final report of the Safe School Initiative: Implications for the prevention of school attacks in the United States is to date the most extensive examination of targeted school violence, including 37 incidents occurring between 1974 and 2000 with altogether 41 attackers [7]. The attackers were all males expressing some major stress prior to the attack, but most of them had not suffered from domestic violence, abuse or serious neglect. The majority had performed academically well and did not have a history of prior violent crimes. Although only 34% of the attackers had received a mental health evaluation and 17% had been diagnosed with a mental health disorder, more than half of them had a history of feeling depressed or desperate prior to the attack. Almost 80% of the attackers had exhibited a history of suicidal ideation or suicidal attempts at some point prior to the attack. Every fourth individual showed a history of alcohol or substance abuse. The motive was revenge in most cases and suicide in one third of the cases. An attempt to get attention or recognition was relevant in approximately 30% of the cases. On the other hand, a lack of guilt, a low capacity for empathy and callous-unemotional traits in adolescence are known to predict the occurrence of criminal behaviour as well as instrumental violence [8], and some of the shooters have been described as having poor anger control, a lack of empathy, features of narcissism as well as feelings of superiority [9]. According to a recent review by Langman [10], the shooters can be categorized as either traumatized, psychotic or psychopathic. Many of the school shooters had been victims of bullying and had suffered from being rejected by their peers [7,11]. They had also experienced a lack of parental supervision and family support as well as troubled family relationships [9].

School shootings are rare occurrences and hard to study, and additional research attention has been needed regarding milder forms of school aggression as well as toward aggressive urges and fantasies [12].

The aim of the present study was to characterize adolescent copycats who had threatened to carry out a school massacre and were sent for psychiatric evaluation for this reason. More accurately, we tried to evaluate if the copycat phenomenon was associated with same features as described among actual school shooters.

## Methods

### Setting

A nation-wide study was conducted on a group consisting of 13-18-year-old adolescents who were sent for adolescent psychiatric evaluation between 8.11.2007 (the day after the first school shooting) and 30.6.2009 because they had threatened to carry out a school shooting. After the threat of school massacre became known, the school and police authorities made the decision to take the adolescent to GP who decided about referring to adolescent psychiatric services. The index event for identifying the adolescent as a subject of the present study was that the referral was registered in specialist level public adolescent psychiatric service.

Information about the study was sent both by e-mail and post to all the chief physicians in the field of adolescent psychiatry in Finland. The chief physicians were asked to go through the referrals made during the above -mentioned time and to select referrals that included threats to carry out a school massacre. Altogether 77 referrals with school massacre threats were found. The chief adolescent psychiatrists were asked to send the basic information of the patients (names and social security numbers) to the researchers. The researchers (N.L. and R.K-H.) traveled to the adolescent psychiatric units and studied the medical files of the index adolescents. The individual persons were not met but the data about them were collected from the files. The study was accepted by the Ethics committee of the Helsinki University Hospital and was approved by the Ministry of Social Affairs and Health.

### Variables

The medical files were retrospectively analysed using a special data collection form. Data on demographics, family- and school related issues, previous psychiatric treatment and previous delinquency were collected.

#### Psychiatric symptoms

Data on the symptoms displayed by the adolescents were collected from the referral and the medical charts written during the assessment and treatment initiated after the threat was made. A total of 21 core symptoms of the adolescent patients were recorded on a checklist (yes/no). All the items of this checklist are seen in Table 1 under the subtitle Current symptoms in the adolescent psychiatric evaluation. The list was originally developed as a screening tool for clinical situations and has been used in research applying retrospective data collection from medical charts [13]. It is not diagnostic instrument and does not attempt to measure an underlying construct, like for example depression rating scales, and thus psychometric properties typically considered in rating scales, such as internal validity, cannot be considered appropriate for this list.

**Table 1:** **Stressful family life events, current psychiatric symptoms, and previous delinquency of the 77 adolescent copycats with schoolshooting threats**

**Stressful life events in family during the last 6 months before the index threat**		
yes	44	57
parental mental disorders	18	23
parental substance use problems	16	21
divorce process	7	9
severe disease of parent/s	7	9
severe problems related to sisters	7	9
domestic violence	4	5
(suspected) sexual abuse	2	3
bereavement	1	1
**Current symptoms in the adolescent psychiatric evaluation**		
symptoms of anxiety	50	65
depressive symptoms	48	62
problems with impulse control	46	60
aggressive outbursts/tantrums	40	52
suicidal ideation	35	45
destroying property	32	42
non-physical aggression towards other people	31	40
violence against other people	31	40
attention problems	30	39
harmful use of alcohol	19	25
isolation	19	25
psychotic symptoms	18	23
truancy/school refusal	9	12
use of illicit drugs	6	8
symptoms of eating disorders	6	8
running away	6	8
suicide attempt	2	3
manic behavior	1	1
property crimes	0	0
inappropriate sexual behavior	0	0
self-harming behaviors	0	0
**Delinquency prior the index threat**		
no	45	58
yes	21	27
pilfering/theft/burglary	15	19
traffic offence	14	18
robbery/assault/attempted homicide	9	12
drug offence	3	4
sex offence	2	3
not clear from files	11	14

#### Adverse family events

With the help of a structured 10-item checklist [13], adverse family life events were recorded. All the items are listed in Table 1 under subtitle Stressful life events in family during the last 6 months before the index threat. Adverse family life events were recorded from referral and/or medical charts written during the assessment and treatment initiated due to the threat. Many of the items dealt with known risk factors for youth violence (see review by Verlinden et al. [9]). The items in the checklist are events that are considered important for adolescent’s well-being in Finnish adolescent psychiatry and they are most likely asked in routine adolescent psychiatric assessment. However it is possible that occasionally an event has occurred even if not recorded in case history. Thus, the numbers describing adverse family life events may be slight underestimates.

#### Psychiatric diagnoses

Psychiatric main diagnoses were collected as given at discharge by the treating psychiatrist according to the ICD-10 [14], which is the official classification used in clinical work in Finland. The diagnoses used in the analyses were classified as follows: mental and behavioural disorders due to psychoactive substance use (F10-19), schizophrenia, schizotypal and delusional disorders (F20-29), mood disorders (F30-39), neurotic, stress-related and somatoform disorders (F40-49), behavioural syndromes associated with physiological disturbances and physical factors (F50-59), disorders of adult personality and behaviour (F60-69), mental retardation (F70-79), disorders of psychological development (F80-89), behavioural and emotional disorders (F90-98) and unspecified mental disorder (F99).

#### The characteristics of the threat expressed and the risk posed by the adolescent

The characteristics of the threat expressed and the risk posed by the adolescent in question was evaluated by an approach presented by Borum and Reddy [15]. In their model, ACTION, a mnemonic guide helps the evaluator to consider the relevant aspects of the motivation, behaviour and attitudes of the potential perpetrator in order to advise for interventions. All available information from the person expressing the threats, the informants close to him/her and their medical files can be used. In the ACTION, mnemonic guide, A (Attitudes) refers to positive attitudes to violence and a commitment to violence-positive ideologies, and the perception that violence is justified in one’s own situation. C (Capacity) refers to the physical and cognitive abilities of the potential perpetrator and his/her potential to fulfill the expressed threat. In T (Thresholds crossed) the evaluator considers whether the person has made preparations to fulfill the threat. Particularly preparations that are in themselves illegal should be considered as signs of increased risk. In I (Intent), intent is assessed: is the person passively attracted to the thought of violence, or actively planning an act? O (Others’ reactions) refers to how those close to the person react to the threats expressed. This includes whether they believe that the person might actually commit the act that s/he is threatening, and also any admiration and support actually expressed or believed to be expressed to the potential perpetrator. N (non-compliance with risk reduction) assesses whether or not the person is motivated to accept interventions that help her/him stay away from violence. The approach is not a structured scale with fixed response alternatives producing a score, but an aid that helps to systematically consider qualitative information and based on this, consider the risk and choose interventions best likely to reduce the risk [15].

#### Psychopathic traits

The Psychopathy Checklist Youth Version (PCL: YV) by Forth et al. [16] was used to assess psychopathic traits. Each of the 20 PCL: YV items is rated either 0 (absent), 1 (present to some degree or contradicting data), 2 (definitely present), or is omitted if the information is insufficient. The total score can range from 0 to 40, with higher scores reflecting a greater number of psychopathic traits. The total PCL score is dimensional, but in research and clinical settings categorical diagnoses are used as well. There is no recommended cut-off score for use with the PCL-YV, but total scores ranging from 30 to 40 are considered diagnostic of psychopathy. A cut-off score of 26 has proven useful in studies performed in Scandinavian countries both among adults [17-20] and adolescents [21], and a score of 20 is sometimes considered to be a cut off for “medium psychopathy” [22]. The items can be summed to yield two factors: factor I or the affective-interpersonal factor (items: impression management, grandiose sense of self-worth, pathological lying, manipulation for personal gain, lack of remorse, shallow affect, callous/lack of empathy, failure to accept responsibility) reflecting the so-called “core psychopathy” and factor II or the behavioral-antisocial factor (items: stimulation seeking, parasitic orientation, poor anger control, early behavioural problems, lacks goals) reflecting an antisocial lifestyle. Although PCL assessments should be based both on a review of file information and a semi-structured interview with the individual, several studies have shown that PCL assessments can reliably be made without an interview when there is sufficient file information available both among adults [23-26] and adolescents [27-31]. The medical files were scored using two officially trained raters who were both adolescent psychiatrists. The individual assessment was rejected from further analysis if it contained more than five omitted items, as instructed in the PCL: YV manual.

#### Comparisons to other Finnish adolescent psychiatric samples

The copycat sample was compared to a sample of adolescent psychiatric inpatients and to a sample of adolescent psychiatric outpatients. The inpatient sample was derived from a research project focusing on involuntary treatment [13]. All the admissions to the adolescent psychiatric wards of Tampere University Hospital in 2004-2006 were identified in hospital databases. Adolescents referred involuntarily for the first time during the data collection period were included in the study as involuntary patients. The next voluntarily referred patient after each involuntary commitment was always included as a control. There were 214 admissions, 106 with voluntary and 108 involuntary referrals, to the study unit in 2004-2006. As each adolescent was included in the study only once, the final study sample comprised 187 adolescent psychiatric patients of whom 93 were referred on an involuntary basis and 94 voluntarily according to the Finnish Mental Health Act. The outpatient sample was primarily collected for a service development project and it focused on lost appointments. The material comprised 99 adolescents who had not shown up for their appointment in the adolescent psychiatric outpatient clinic in Tampere University Hospital in August-September 2007, and always the next adolescent who had an appointment to the same clinical worker (doctor, psychologist, social worker or nurse) and showed up as expected (n = 99). No show was defined as not showing up without any notice, or as cancelling just before the appointment time. The adolescents were identified in the hospital database where information is routinely given about the details of each appointment, including no show and cancellations. In both projects, information on symptoms and adverse life events were collected similarly as in the present copycat data.

### Statistics

The study is primarily descriptive. The psychiatric symptoms and adverse family life events among the copycats were compared to symptoms and family adversities among 1) Finnish adolescent psychiatric inpatients, and 2) Finnish adolescent psychiatric outpatients. First, cross-tabulations with chi-square statistics were carried out. To control for confounding by age and sex, logistic regression was used. Each symptom in the symptom checklist, and each family adversity, was entered in turn as the dependent variable. Age (continuous), sex and sample (first, copycats vs. inpatients; second, copycats vs. outpatients) were entered as the independent variables. OR: s with 95% confidence intervals are reported.

## Results

### Background, symptoms and previous delinquency

All of the copycats were Caucasian native Finns with mean age of 15.0 years (SD 1.48, range 13-18 years). Most of them (87%, n = 67) were males. 90% of them (n = 69) were living with one or both biological parents, 9% (n =7) in an institution and 1% (n = 1) without a guardian. They were all still registered in school: a majority of them went to comprehensive school (75%, n = 56), and the rest to vocational school (14% [n = 11]) or upper secondary school (13% [n = 10]). Of them, 66% (n = 56) were performing well or on average level at school, and 34% (n = 26) were academically behind the age group or currently discontinued school.

Of the copycats, 57% (n = 44) had had a contact to child (42%, n = 32) or adolescent (16%, n = 12) psychiatric services prior to the index contact. Previous diagnoses had been set behavioral and emotional disorders (F90-99) (25%, n = 19), mood disorders (F30-39) (14%, n = 11), disorders of psychological development (F80-89) (12%, n = 9) and schizophrenia group diagnoses (F20-29) (6%, n = 5). 27% (n = 21) had been clients of child welfare before the index threat.

More than half of the copycats had experienced stressful events in their family life within the last six months before the index threat with the most prevalent stressors being a mental disorder and/or the alcoholism of one or both parents (see Table 1). Of them, 39% (n = 30) reported that they were victims of bullying at school.

More than half of the copycats did not show previous delinquency. Among those with previous criminal acts, the most prevalent types of crime were petty theft, theft and burglary as well as traffic offences (see Table 1).

The current psychiatric symptoms of the copycats are shown in Table 1. Almost two thirds of the copycats suffered from anxiety and depressive symptoms, and almost half of the sample expressed either suicidal ideation or suicidal plans. Behavioural problems, including impulse control problems, aggressive outbursts, the destruction of property as well as non-physical and physical violence against other persons, were common. Approximately every fourth individual expressed psychotic symptoms as well as isolation.

The prevalence of current clinical diagnoses was high, as seen in Table 2. The diagnosis groups highlighted were behavioural and emotional disorders, mood disorders, disorders of psychological development as well as schizophrenia-related disorders. Sixty percent of the copycats with disorders of psychological development suffered from pervasive developmental disorders (F84).

**Table 2:** Primary clinical ICD-10 diagnoses in copycat adolescents with school massacre threats (n = 77)

	**n**	**%**
F0-09 Organic, including symptomatic, mental disorders	0	0
F10-19 Mental and behavioral disorders due to psychoactive substance use	0	0
F20-29 Schizophrenia, schizotypal and delusional disorders	9	12
F30-39 Mood disorders	16	21
F40-49 Neurotic, stress-related and somato form disorders	8	10
F50-59 Behavioral syndromes associated with physiological disturbances and physical factors	2	3
F60-69 Disorders of adult personality and behavior	3	4
F70-79 Mental retardation	1	1
F80-89 Disorders of psychological development	13	17
F84 Pervasive developmental disorders	8	10
F90-98 Behavioral and emotional disorders	18	23
F99 Unspecified mental disorder	1	1
No psychiatric diagnosis	6	8

### The threat expressed

The school massacre threats were expressed orally in most cases (see Table 3).

**Table 3:** Characteristics of the index school massacre threat in 77 adolescent copycats

	**n**	**%**
**How the index threat was expressed**		
orally expressed to a teacher/school friends/therapist	43	56
in a letter/note/essay/exam paper	17	22
via Internet	17	22
**A - Action**		
**Positive attitudes towards aggressive behavior in general**		
yes	58	75
not clear from files	12	16
no	7	9
**Positive attitudes towards previous school shootings**		
yes	43	56
no	19	25
not clear from files	15	19
**Felt justification for the attack**		
yes	58	75
not clear from files	15	19
no	4	5
**Motive**		
revenge against identified persons	34	44
anger and hatred in general	27	35
desire to die (homicide-suicide fantasy)	24	31
wanting attention	4	5
joke	3	4
unclear	2	3
**C – Capacity to fulfill the threat**		
yes	42	55
not clear from files	18	23
no	17	22
**T – Thresholdscrossed**		
no preparations made	46	60
preparations made	20	26
not clear from files	11	14
**I - Intention for the index threat**		
passive thinking	56	73
clear intention	14	18
not clear from files	7	9
**O – Others’ reactions**		
parents took the threat seriously	38	49
parents did not take the threat seriously/disparaged the threat	19	25
parents were ambivalent	18	23
not clear from files	2	3
**N – Non-compliance to risk reaction**		
the adolescent was against psychiatric evaluation/treatment	44	57
the adolescent was ambivalent to psychiatric evaluation/treatment	17	22
the adolescent was neutral or positive to psychiatric evaluation/treatment	16	21
not clear from the files	0	0

Most of the copycats expressed positive attitudes towards aggressive behaviour in general and more than half of them towards the former school shooters. Most of the copycats felt that there was justification for a violent attack with the most prevalent motive being revenge against certain persons. One third of the copycats expressed the idea of extended suicide. Only a few explained that the threat was a joke or an attempt to get attention. (see Table 3).

More than half of the adolescents were estimated to have the capacity to fulfill the threat (see Table 3). However, in most cases the intention to carry out the index threat was low and remained on the level of passive thinking. Approximately every fifth individual had made preparations to carry out the threat (see Table 3).

Nearly half of the parents took the massacre threat seriously, but one fourth of the families did not take the threat seriously or disparaged it (see Table 3).

More than half of the adolescent did not want to participate in the psychiatric evaluation and treatment offered, but approximately every fifth adolescent expressed neutral or positive feelings about psychiatric interventions (see Table 3).

### Psychopathic traits

Out of the 77 PCL:YV assessments, 27 were rejected from the statistical analysis due to having more than five omitted items. Only one of the copycats was assessed as expressing high traits of psychopathy. The mean PCL-YV total and factor scores are presented in Table 4.

**Table 4:** Psychopathy Check List- youth version (PCL-YV) mean (SD) total scores and factor scores of the copycat adolescents with school massacre threats (n = 50)

	**mean**	**SD**	**range**
PCL-YV total score	6.5	5.30	0-26
PCL-YV factor I (Affective-Interpersonal)	2.3	2.3	0-8
PCL-YV factor II (Lifestyle-Antisocial)	3.7	3.4	0-14
	**n**	**%**	
PCL-YV total score ≥ 30	0	0	
PCL-YV total score 20-29	1	2	
PCL-YV total score 10-19	11	22	
PCL-YV total score 1-9	31	62	
PCL-YV total score 0	7	14	

### The copycats as compared to previous Finnish clinical adolescent psychiatric samples

The copycats displayed most of the studied symptoms as commonly as the adolescents in the comparison inpatient and outpatients samples. They had more frequently shown temper tantrums, breaking property, impulse control problems and anxiety than the inpatients and the outpatients, and as compared to inpatients, also attention problems were more common in the copycats. They displayed less commonly psychotic symptoms and manic behaviors than the inpatients, less often and truancy/school refusal than the inpatients and the outpatients (see Table 5).

**Table 5:** Prevalence (% [n/N]) of emotional and behavioural symptoms among the copycats and in a sample of adolescent psychiatric inpatients and one of outpatients, and risk (OR, 95% confidence intervals) for emotional and behavioural symptoms according to sample (inpatients vs. copycats, and outpatients vs. copycats), controlled for age and sex

**Symptom**	**Copycats**	**Inpatients**	**Outpatients**	**Inpatients vs. Copycats**		**Outpatients vs. Copycats**	
				**p**	**OR (95% Cl)**	**p**	**OR (95% Cl)**
suicidal ideation & talk	45.5 (35/77)	63.1 (118/187)	34.8 (69/198)	0.006	1.5 (0.8-2.9)	0.07	0.4 (0.12- 0.7)
suicide attempt	2.6 (2/77)	19.8 (37/187)	2.5 (5/198)	<0.001	3.8 (0.8-17.0)	0.62	0.7 (0.09-5.4)
self-harming behaviours	-	40.1 (78/187)	23.2 (46.198)	<0.001	n.a.	<0.001	n.a.
psychotic symptoms	23.4 (18/77)	48.7 (91/187)	23.7 (47/198)	<0.001	**2.2 (1.1- 4.3)**	0.47	0.6 (0.3-1.3)
depression	62.3 (48/77)	74.9 (140/187)	56.9 (118/198)	0.03	1.5 (0.8-2.8)	0.42	0.5 (0.3-1.0)
manic behaviour	1.3 (1/77)	6.4 (12/187)	0.5 (1/198)	**0.07**	**10.7 (2.2- 98.8)**	0.48	0.2 (0.003-8.2)
hostile behaviour	40.3 (31/77)	26.7 (50/187)	12.6 (25/198)	0.02	1.0 (0.5-1.9)	<0.001	0.6 (0.3-1.3)
temper tantrums	51.9 (40/77)	15.0 (28/187)	9.1 (18/198)	**<0.001**	**0.3 (0.1-0.5)**	**<0.001**	**0.2 (0.08-0.4)**
violent behaviour	40.3 (31/77)	28.3 (53/187)	14.1 (28/198)	0.04	1.1 (0.6-2.1)	<0.001	0.6 (0.3-1.2)
breaking property	41.6 (32/77)	10.2 (19/186)	7.6 (15/198)	**<0.001**	**0.3 (0.1-0.6)**	**<0.001**	**0.3 (0.1-0.7)**
inappropriate sexual behaviour	-	8.0 (15/187)	10.1 (20/198)	0.03	n.a.	0.001	n.a.
alcohol abuse	24.7 (19/77)	32.1 (60/187)	28.8 (57/198)	0.22	1.4 (0.7-2.8)	0.32	1.0 (0.5-2.2)
substance use	7.8 (6/77)	13.9 (26/187)	7.6 (15/198)	0.12	1.0 (0.3-3.1)	0.55	0.7 (0.2-2.4)
truancy/school refusal	11.7 (9/77)	36.9 (69/187)	30.3 (60/198)	**<0.001**	**5.7 (2.5- 13.1)**	**0.001**	**3.0 (1.2- 7.3)**
property crimes	-	9.6 (18/187)	6.6/13/198)	0.002	n.a.	0.01	n.a.
eating disorder symptoms	7.9 (6/77)	23.5 (44/187)	26.8 (53/198)	0.002	1.6 (0.6-4.5)	<0.001	1.2 (0.4-3.8)
isolation	24.7 (19/77)	5.3 (10/187)	20.2 (40/198)	<0.001	1.1 (0.4-1.3)	0.24	0.8 (0.4-1.7)
impulse control problems	59.7 (46/77)	3.2 (6/187)	21.7 (24/198)	**<0.001**	**0.03 (0.01-0.08)**	**<0.001**	**0.4 (0.2-0.8)**
running away	7.8 (6/77)	13.9 (26/187)	7.1 (14/198)	0.12	1.4 (0.5-4.1)	0.50	1.2 (0.4-4.3)
attention problems	39.0 (30/77)	0.5 (1/187)	26.3 (52/198)	**<0.001**	**0.004 (<0.001-0.04)**	0.02	1.0 (0.5-2.0)
anxiety	64.9 (50/77)	9.6 (18/187)	61.6 (122/198)	**<0.001**	**0.03 (0.01-0.09)**	**0.31**	**0.3 (0.1-0.6)**

n.a. = notapplicable.

As to adverse family life events, the inpatients had more frequently experienced family violence and bereavement than the copycats, whereas the copycats had more often parent(s) with severe mental disorder. From the comparison outpatient sample the copycats only differed in having less often experienced family violence (see Table 6).

**Table 6:** Prevalence (% [n/N]) of adverse family life events or conditions among the copycats, in a sample of adolescent psychiatric inpatients and an outpatients, and risk (OR, 95% confidence intervals) of adverse family life events according to sample (inpatients vs. copycats and outpatientsvs.copycats.), controlled for age and sex

**Symptom**	**Copycats**	**Inpatients**	**Outpatients**	**Inpatients vs. Copycats**		**Outpatients vs. Copycats**	
				**p**	**OR (95% CI)**	**p**	**OR (95% CI)**
family violence	5.2 (4/77)	16.6 (31/187)	16.7 (33/198)	**0.009**	**3.8 (1.2- 12.3)**	**0.008**	**3.9 (1.1- 13.7)**
parental substance use problems	20.8 (16/77)	17.1 (32/187)	12.5 (24/198)	0.28	0.6 (0.3-1.4)	0.05	0.64 (0.26- 1.60)
divorce or separation	9.1 (7/77)	5.9 (11/187)	6.1 (12/198)	0.24	0.6 (0.2-1.9)	0.25	0.49 (0.13- 1.79)
bereavement	1.3 (1/77)	12.8 (24/187)	8.1 (16/198)	**0.002**	**10.5 (1.3- 84.4)**	0.03	7.89 (0.88- 68.4)
parental severe somatic illness	9.1 (7/77)	3.7 (7/187)	9.1 (18/198)	0.07	0.5 (0.1-1.8)	0.57	0.80 (0.24- 2.65)
parental severe mental disorder	23.4 (18/77)	11.8 (22/187)	15.2 (30/198)	**0.03**	0.4 (0.2-1.0)*	0.11	0.84 (0.36- 1.98)
severe financial difficulties, unemployment etc.	10.4 (8/77)	4.8 (9/187)	12.1 (24/198)	0.08	0.5 (0.2-1.6)	0.45	0.58 (0.19- 1.77)
severe problems related to siblings	9.1 (7/77)	7.0 (13/187)	15.2 (30/198)	0.35	0.5 (0.2-1.6)	0.14	0.82 (0.27- 2.52)
(suspected) sexual abuse within the family	2.6 (2/77)	3.7 (7/187)	2.5 (5/198)	0.49	0.5 (0.1-3.9)	0.62	0.30 (0.03-2.88)

*p = 0.04.

## Discussion

Juvenile delinquency is linked to psychiatric morbidity [32,33]. In the present sample the prevalence of mental and behavioural disorders was considerably high. According to community-based studies, the prevalence of mental health disorders in adolescence is approximately 15-25% [34]. Aggressive behaviour either towards others or towards oneself, has been associated with depressive disorders [35], conduct disorders [36,37], schizophrenia- related disorders [38-40] as well as pervasive developmental disorders [41-43]. In the present sample, almost two thirds of the copycats suffered from depressive symptoms, almost half of the sample expressed either suicidal ideation or suicidal plans, and every fifth adolescent was diagnosed with clinical depression. The prevalence of depression in adolescent community samples has been estimated to be approximately 5-10% [44,45]. According to a study by Pelkonen and Marttunen [35], 20-25% of 13- to 16-year-old Finnish girls and 15% of boys had considered suicide during the previous year. In the present sample, 25% of the adolescents were suffering from contact disorder, and the prevalence of various externalizing symptoms was much higher. According to community-based studies, the prevalence of conduct disorder in adolescence is approximately 5-10% [46,47]. According to Finnish studies, estimates of antisocial behavior have ranged between 12.9 and 29.4% among 14- to 16-year-old adolescents [48,49]. The prevalence of schizophrenia-related disorders in adolescence has been estimated to be 1-2% [34,50], but in the present sample it was 12%. According a recent study by Fernell and Gillberg [51], the prevalence of pervasive developmental disorders in the population is 0.6-1%, and among copycats it was 10%. Overall, adolescents expressing threats to carry out a school massacre appear to be severely disturbed, and referral to adolescent psychiatric services in these situations is justified.

The copycats displayed more symptoms illustrative of aggression control problems and anxiety than adolescent psychiatric inpatients and outpatients, and differed from the inpatients also by presenting more often with attention problems. They displayed psychotic symptoms and manic behaviors less often than the inpatients but as often as the outpatients. Regarding most of the studied emotional and behavioural symptoms, the copycats were as disturbed as the inpatients and the outpatients. Their need of psychiatric attention is thus as strong as of those who have been taken into outpatient or inpatient care, and both emotional and behavioral symptoms need to be taken into account in the treatment plan. Aggression management needs are emphasized. However it is also noticeable that the copycats had actually not acted violently against other people more frequently than adolescent psychiatric outpatients or inpatients.

A subtype of conduct disorder has been described in which the adolescent lacks a sense of guilt, has a low capacity for empathy, manipulates others and is callous and unemotional [52]. The youngsters with these psychopathic traits have more severe and pervasive behavioural problems than other conduct- disordered adolescents. They are also more trill seeking [52], more reactive to rewards than punishments [53] and are more inclined to associate positively with violent behaviour [54]. In two community samples of altogether 160 male adolescents, the mean PCL: YV total scores varied between 2.85 and 3.98 [16]. In file-based PCL: YV assessments among delinquent adolescents, the mean total scores have varied between 22.8- 23.8 [29,31]. As a group the copycats scored somewhat higher than adolescents in previous community samples, but they did not show the considerable affective-interpersonal features of psychopathy nor the antisocial lifestyle typical of forensic samples.

Even though most of the copycats were living with both their parents, as is the case among the same-aged population at large in Finland [55], adverse family life events were common among them. Adverse family life events are likely to result in reduced parental supervision and attention, which in turn are likely to promote the development of problems in the adolescent offspring [56]. Compared to adolescent psychiatric inpatients, parental mental disorders were more common among the copycats [13]. This may suggest a particular vulnerability to the development of mental disorders in circumstances perceived as stressful. In promoting behavioural control among adolescents who express threats of carrying out a school massacre or similar, particular attention needs to be paid to the parental capacity to support positive adolescent development and the parents` own mental health needs.

Bullying has been widely recognized as a societal problem and is an issue of widespread concern. Findings concerning the increased risk of victims of bullying later indulging in violent behaviour have been contradictory. In a study using the police register of alleged offences, victims of bullying had approximately a two- to threefold higher risk of committing a crime than adolescents not involved in bullying behaviour, although this increased risk existed only in the presence of psychiatric symptoms [57]. In a recent study by Luukkonen et al. [58] using official criminal records, being a victim of bullying was, however, not a risk factor for later criminality among hospital-admitted adolescents. In a large Finnish national school health survey among 8th and 9th-grade comprehensive school students in 2007, 10% of the boys and 6% of the girls reported being victims of bullying at least once a week [59]. In the present sample, the prevalence of being a victim of bullying was considerably larger, as almost 40% of the copycats reported being bullied.

The need to belong has been described as a powerful, fundamental and extremely pervasive motivation [60]. Aggression is the most common reaction to peer rejection [61]. The high prevalence of bullying, which definitely can be seen as a form of rejection, might explain the finding that revenge was the most frequent motive for massacre threats. We could not confirm whether the copycats had actually been bullied, but it is exactly the perception of being bullied that is likely to push an individual´s reaction towards hatred and the need for revenge. The idea of homicide-suicide was surprisingly common, since extended suicides have been reported to be rare among young people [62]. Joking or wanting attention were mentioned as motives very seldom, which underlines the fact that the threats of the copycats must be taken seriously and should always be more closely examined.

Positive attitudes to violence have been shown to be an important violence risk factor in adolescence [63]. Among the copycats, a positive attitude to violent solutions was common. Despite the fact that Finland is a welfare state with a strong social security system, equalizing educational, health and social policies, and a low threshold of access to social and health services, the society is also violent. Suicide rates are among the highest in Western Europe (http://www.who.int/mental_heallth/prevention/suicide/suiciderates/en/), as are also the homicide rates among adults, if however not among adolescents [64]. Expressing threats of violence towards public figures is commonplace. After the two school massacres, empathy and admiration towards the perpetrators was expressed in certain public discussions, particularly on the Internet. Recent political developments have also suggested increasing acceptance towards racist hostility, which is a further indication of positive attitudes towards violence in society, even though racial issues did not emerge in the present sample. Adolescent violent crime has not shown a similar decreasing trend as property offences, and population studies do not suggest an increase in adolescent violent crime, either [65].

In line with The final report of the Safe School Initiative: Implications for the prevention of school attacks in the United States [7], the copycats were mostly boys, who performed academically well and did not have a history of prior violent crimes, but who expressed depressive symptoms, suicidal ideation as well as harmful use of alcohol. Langman [10] characterized school shooters as either psychotic, psychopathic or traumatized. Our sample of copycats displayed less psychotic symptoms than Finnish adolescent psychiatric inpatients, but nevertheless far more psychotic symptoms than adolescent population [66], and the prevalence of schizophrenia group psychoses was among them more than 10-fold as compared to populations. The copycats bear a resemblance to school shooters in being psychotic. Psychopathic traits were rare in our sample of copycats. As to being traumatized, experience of being bullied emerged as a strong characteristic of the copycats, even if they were less traumatized than adolescent psychiatric inpatients regarding family adversities. Our data does not reveal whether the copycats really had been victimized by their peer or whether their experience of being bullied related to distorted perception of social interaction. However, the subjective experience of being victimized may influence a person’s emotional and behavioural balance more than the actual nature of interactions [67]. In sum, the copycats seemed to resemble in many ways the actual school shooters. However, although making threats is unacceptable behaviour, one must at the same time keep in mind that it is not the same as realized homicide. The relevant question for future research is which factors distinguish the adolescents who make threats from those who carry them out. The role of protective factors might be extremely important to study.

The strength of this study was its nationwide nature. However, the fact that the study was retrospective and register based does present some obvious limitations. The diagnoses were not based on structured interviews, but were taken from patient medical files. In this regard, in Finland the basic diagnostic procedures have been proven reliable [68,69]. The study was descriptive in nature. The quality of the medical files varied, and the data concerning family life, school as well as previous criminality was based on information given by the adolescents and their guardians, teachers and social workers. The official files from child welfare services or criminal records (note: in Finland, the minimum age of criminal liability is 15 years) were not used. We collected variables that are typically investigated in Finnish adolescent psychiatric examination. The items in the checklist are events that are considered important for adolescent’s well-being in Finnish adolescent psychiatry and they are most likely asked in routine adolescent psychiatric assessment. However it is possible that occasionally an event has occurred even if not recorded in case history. Because of this, the psychopathology found in the present sample is rather underestimated than overestimated. Unfortunately, it was not possible to find the total number of school massacre threats expressed during the study period, but the number has been estimated to be in the hundreds, according to the Finnish police (see the Introduction). It is likely that the sample of the present study consisted of those adolescents of whom the school and social authorities had been the most concerned regarding their mental health and, because of this, generalizing the results to other copycats, who might even display more delinquent features must be done with caution.

## Conclusion

The copycats with school massacre threats were characterized with a high prevalence of mental and behavioural disorders. Like actual school shooters, they showed psychotic symptoms and traumatic experiences, but unlike the shooters, the copycats were not psychopathic.

## Competing interests

The authors declare that they have no competing interests.

## Authors’ contributions

NL reviewed the medical files, scored the PCL-YV, organized the data and served as the first author. ES analysed the data and participated in the writing process. RK-H reviewed the medical files, scored the PCL-YV and participated in the writing process. All authors read and approved the final manuscript.

## Pre-publication history

The pre-publication history for this paper can be accessed here:

http://www.biomedcentral.com/1471-244X/12/91/prepub
